# Molecular Profiling: A Case of* ZBTB16-RARA* Acute Promyelocytic Leukemia

**DOI:** 10.1155/2017/7657393

**Published:** 2017-04-26

**Authors:** Stephen E. Langabeer, Lisa Preston, Johanna Kelly, Matt Goodyer, Ezzat Elhassadi, Amjad Hayat

**Affiliations:** ^1^Cancer Molecular Diagnostics, St. James's Hospital, Dublin 8, Ireland; ^2^Department of Clinical Genetics, Our Lady's Children Hospital, Dublin 12, Ireland; ^3^Department of Haematology, Galway University Hospital, Galway, Ireland

## Abstract

Several variant* RARA* translocations have been reported in acute promyelocytic leukemia (APL) of which the t(11;17)(q23;q21), which results in a* ZBTB16-RARA* fusion, is the most widely identified and is largely resistant to therapy with all-trans retinoic acid (ATRA). The clinical course together with the cytogenetic and molecular characterization of a case of ATRA-unresponsive* ZBTB16-RARA* APL is described. Additional mutations potentially cooperating with the translocation fusion product in leukemogenesis have been hitherto unreported in* ZBTB16-RARA* APL and were sought by application of a next-generation sequencing approach to detect those recurrently found in myeloid malignancies. This technique identified a solitary, low level mutation in the* CEBPA* gene. Molecular profiling of additional mutations may provide a platform to individualise therapeutic management in patients with this rare form of APL.

## 1. Introduction

Acute promyelocytic leukemia (APL) is a distinct form of acute myeloid leukemia (AML) characterized by the balanced translocation t(15;17)(q24;q21) that results in production of the* PML-RARA* oncogene. Retinoid-based therapy in combination with either anthracyclines or arsenic trioxide results in favorable rates of complete remission and overall survival, provided that the patient can be supportively managed through the initial coagulopathy [1]. However, a small proportion of patients harbor variant translocations that result in fusion of* RARA* to one of a number of alternative partner genes [2, 3]. The most often reported of these variant translocations is the t(11;17)(q23;q21) which results in the fusion of the zinc finger gene* ZBTB16* (formerly* PLZF*) to the* RARA* locus [4, 5]. The bone marrow morphology of patients with* ZBTB16-RARA* APL tends to be distinct from those patients with either classical or the hypogranular variant of APL [6]. Identification of the* ZBTB16-RARA* fusion is critical for therapeutic purposes as these patients are generally resistant to differentiating retinoid therapy, specifically all-trans retinoic acid (ATRA) [7], and treatment should be with standard AML regimens according to current recommendations [8].

In murine models of APL, induction of* PML-RARA* expression in myeloid stem cells results in a myeloproliferative disease that subsequently develops into leukemia with promyelocytic features after a relatively long latency implicating the requirement for further cooperating mutations to fully recapitulate the APL phenotype [9, 10]. The development and application of whole exome sequencing and targeted exome sequencing have led to the identification of several cooperating mutations in APL and demonstrates the clonal and subclonal acquisition of mutational events in conjunction with the driver* PML-RARA* oncogene [11, 12]. A similar pattern of these cooperative mutations appears to exist within patients with APL and those with other types of AML [13]. Whether these additional mutations have a prognostic impact is unclear [14]. However, identification of these mutations may allow targeted intervention [15]. To date, the pattern of cooperating mutations in patients with* ZBTB16-RARA* APL has not been investigated. Characterization of a patient with this uncommon cytogenetic variant of APL is described with subsequent application of a targeted next-generation sequencing (NGS) approach to identify allied mutational events.

## 2. Case Report

**Figure 1:**
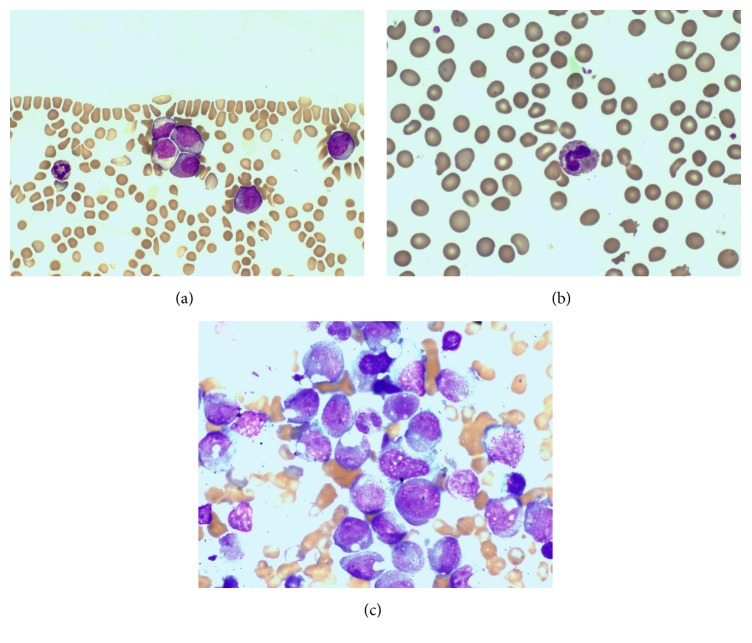
(a) Peripheral blood promyelocytes; (b) peripheral blood pseudo-Pelger-Huet neutrophils; (c) bone marrow effacement by promyelocytes at diagnosis.

An 81-year-old female presented with a short history of back pain with a physical examination proving unremarkable. Full blood count revealed pancytopenia (hemoglobin 8.8 g/dl, neutrophils 1.8 × 10^9^/L, and platelets 140 × 10^9^/L) and abnormal circulating promyelocytes that constituted 40% of nucleated cells. A coagulation screen was only mildly deranged with a prothrombin time of 15.1 seconds, an activated partial thromboplastin time of 29.4 seconds, and fibrinogen of 3.2 g/L. Peripheral blood promyelocytes, which accounted for 40% of nucleated cells, had regular nuclei, were hypergranular, and lacked Auer rods (Figure 1(a)) with increased numbers of pseudo-Pelger-Huet neutrophils noted (Figure 1(b)). Immunophenotyping detected autofluorescent blasts that expressed MPO, CD13, CD14, and CD33 but lacked CD34 and HLA-DR expression. The bone marrow was effaced by promyelocytes (Figure 1(c)) which stained strongly positive for Sudan Black. The patient commenced ATRA with a progressive concurrent increase noted in both peripheral blood myeloblasts and promyelocytes (white cell count 40.5 × 10^9^/L), thrombocytopenia (platelets 92 × 10^9^/L), and coagulopathy (fibrinogen 0.7 g/L) despite intensive blood product support. The chest findings were more consistent with aspiration and therefore no response to ATRA with differentiation syndrome excluded. Hydroxyurea and idarubicin were initiated in an attempt to achieve cytoreduction; however, progressive respiratory compromise and worsening coagulopathy led to the patients' death due to pulmonary hemorrhage ten days after presentation.

**Figure 2:**
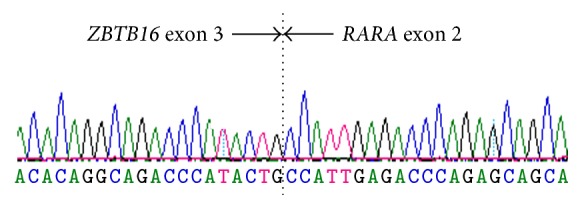
Sanger sequencing demonstrating fusion of* ZBTB16* exon 3 to* RARA* exon 2.

Cytogenetic G-band analysis identified an aberration involving 17q in 4/13 metaphases analysed. Metaphase fluorescent in situ hybridisation studies revealed a rearrangement involving* RARA* at 17q12 and confirmed the partner chromosome to be 11q, thus identifying the t(11;17) translocation with a full karyotype of 46,XX,add(17)(q21)[4]/46,XX[9].ish der(11)t(11;17)(q23;q21)(RARA+).nuc ish(PMLx2,RARAx3)[142/200]. A standardised reverse transcription-PCR approach [16] did not detect* PML-RARA* transcripts, but, using primers previously described [7], a* ZBTB16-RARA* fusion transcript, consistent with fusion of* ZBTB16* exon 3 fused to* RARA* exon 2, was detected and confirmed by Sanger sequencing (Figure 2). The reciprocal* RARA-ZBTB16 *fusion was not detected.

An NGS approach utilising a gene panel to detect additional mutations cooperating with the* ZBTB16-RARA* fusion in propagating APL was retrospectively employed. Amplicon libraries covering thirty commonly mutated genes implicated in myeloid malignancies were generated using genomic DNA from the diagnostic bone marrow aspirate using an Ion AmpliSeq™ approach (Thermo Fisher Scientific, Paisley, UK). A single in-frame* CEBPA* p.P189delP mutation was detected (c.564–566 delGCC; reference sequence GRCh37: 19:33792755-7) with an allele frequency of 6.2%.

## 3. Discussion

Several rare variant* RARA* translocations have been described in APL among which the* ZBTB16-RARA *is the most frequent. Identification of the t(11;17) at the cytogenetic and/or the* ZBTB16-RARA* fusion at the cytogenetic and molecular levels, respectively, is paramount as, in the case described herein,* ZBTB16-RARA* APL is generally unresponsive to ATRA therapy, although it must be noted that some response to initial ATRA therapy has been documented in very rare cases and always in combination with other agents [17–19]. Although distinctive morphological features have been ascribed to* ZBTB16-RARA* APL [6], the peripheral blood and bone marrow morphology in this case more closely resembled that of typical APL, an aspect infrequently reported [20].

The NGS approach adopted herein detected only one additional mutation in* CEBPA* which encodes a transcription factor involved in cell fate decisions for myeloid cell differentiation [21]. This gene is recurrently mutated in types of AML other than APL, but mutations at this codon have been rarely documented [22]. Although no constitutional material was available and therefore not analysed in tandem, the low allele frequency implies that this is an acquired, somatic mutation of* CEBPA* and not a single nucleotide polymorphism. How* CEBPA* mutations might cooperate with the* ZBTB16-RARA* fusion in leukemogenesis is unclear; however, one study has demonstrated that CEBPA protein activity is severely impaired in leukemic promyelocytes with the t(11;17) translocation and that the reciprocal RARA-ZBTB16 protein inhibits myeloid cell differentiation through its interaction with CEBPA [23]. It is acknowledged that this NGS approach is limited to those commonly mutated genes in myeloid malignancies and that a greater coverage of both other genes and additional exons of those genes within this panel may prove more informative.

In conclusion, rapid identification and comprehensive molecular profiling are vital for future appropriate tailoring of therapy in patients with this rare form of APL.
